# Control and Monitoring of Milk Renneting Using FT-NIR Spectroscopy as a Process Analytical Technology Tool

**DOI:** 10.3390/foods8090405

**Published:** 2019-09-12

**Authors:** Silvia Grassi, Lorenzo Strani, Ernestina Casiraghi, Cristina Alamprese

**Affiliations:** Department of Food, Environmental, and Nutritional Sciences, Università degli Studi di Milano, via G. Celoria 2, 20133 Milan, Italy

**Keywords:** dairy industry, milk renneting, in-line control, near infrared spectroscopy, MCR-ALS, multivariate control chart

## Abstract

Failures in milk coagulation during cheese manufacturing can lead to decreased yield, anomalous behaviour of cheese during storage, significant impact on cheese quality and process wastes. This study proposes a Process Analytical Technology approach based on FT-NIR spectroscopy for milk renneting control during cheese manufacturing. Multivariate Curve Resolution optimized by Alternating Least Squares (MCR-ALS) was used for data analysis and development of Multivariate Statistical Process Control (MSPC) charts. Fifteen renneting batches were set up varying temperature (30, 35, 40 °C), milk pH (6.3, 6.5, 6.7), and fat content (0.1, 2.55, 5 g/100 mL). Three failure batches were also considered. The MCR-ALS models well described the coagulation processes (explained variance ≥99.93%; lack of fit <0.63%; standard deviation of the residuals <0.0067). The three identified MCR-ALS profiles described the main renneting phases. Different shapes and timing of concentration profiles were related to changes in temperature, milk pH, and fat content. The innovative implementation of MSPC charts based on T^2^ and Q statistics allowed the detection of coagulation failures from the initial phases of the process.

## 1. Introduction

Milk coagulation is one of the most critical steps during cheese manufacturing. Failures in this operation can lead to decreased yield, anomalous behaviour of cheese during storage, significant impact on cheese quality and process wastes. Until recently, curd formation progress and cutting time setting have been mainly managed by skilled personnel through process variable control (e.g., vat temperature, rennet activity and concentration, calcium salt concentration, pH) [1]. However, systems for designing, analysing, and controlling manufacturing processes through timely measurements are spreading rapidly among the dairy industries [2]. These systems fall under the Process Analytical Technology (PAT) principles, borrowed from the pharmaceutical industry, which aimed to answer the challenge of ensuring final product quality by real-time process monitoring [3].

The PAT approach requires process analysers to be implemented in dynamic conditions. For the dairy industry, in-line mechanical and/or optical devices have been proposed instead of the gold standard lactodynamograph based on the oscillation recording of a small stainless-steel pendulum immersed in milk [2]. For instance, acoustic wave sensors [4], small angle neutron scattering techniques [5], and ultrasonic analyses [6,7] have been evaluated. Spectroscopic sensors are among the most promising process analysers in food industry [8] and different PAT solutions have been proposed for their implementation in cheese manufacturing [9]. For instance, fluorescence spectroscopic sensors have been widely used to study and monitor rennet coagulation of milk [10], also assessing coagulum strength and gelation time [11]. However, near-infrared (NIR) spectroscopy presents numerous advantages compared to other spectroscopic techniques. In fact, NIR spectroscopy has been demonstrated to be suitable for fast, non-destructive, and low-invasive real-time measurements of both quality parameter evolution [12] and process dynamics [13,14], due to the possibility to extract both chemical and physical information from a NIR spectrum. Moreover, NIR measurements can be realized with ad hoc optic probes placed directly into the coagulation vats [14,15,16,17,18].

After the proper process analyser implementation, PAT requires robust data management and analysis tools for providing platform solutions [19]. In particular, chemometrics can be applied to handle spectroscopic data for process monitoring, control, and endpoint determination, by replacing univariate and bivariate statistical techniques. For instance, Multivariate Curve Resolution (MCR) was applied to elucidate process-related physico-chemical changes and to extrapolate process kinetic information. Indeed, MCR infers the contribution of each single component involved in the studied system, allowing its quantification over the process development [20]. Its successful application in the spectroscopic field is due to the ability in decomposing spectroscopic data that are characterized by overlapped spectral bands, especially when recorded from complex systems such as milk [18,21].

The existing PAT approaches for milk renneting monitoring are able to detect the occurrence of coagulation point or to evaluate the curd setting rate, but they assume kinetic models on a case-by-case basis [2]. Moreover, the existing models often study reconstituted milk from skim milk powder and do not consider process variables such as coagulation temperature, or milk pH and/or fat content, which can vary depending on the cheese to be produced.

In this context, the present study proposes a PAT approach for milk renneting monitoring based on Fourier Transform (FT)-NIR spectroscopy coupled with MCR-ALS. A wide range of normal operating conditions (NOC) adopted for cheese production have been considered in order to make the proposed models more robust. Through this approach, Multivariate Statistical Process Control (MSPC) charts have been implemented for fault diagnosis and ongoing process management, no matter the applied operating conditions. The development of the MSPC charts represents an important innovation since, to the best of our knowledge, in the scientific literature there are not works dealing with multivariate control charts implemented for milk rennet coagulation.

## 2. Materials and Methods 

### 2.1. Experimental Plan

Twelve milk renneting batches (named from NOC_1_ to NOC_12_) were set up under different normal operating conditions commonly applied in cheese production, in order to describe process changes due to different industrial settings.

In particular, three levels of coagulation temperature (30 °C, 35 °C, 40 °C), milk pH (6.3, 6.5, 6.7) and fat content (0.1 g/100 mL, 2.55 g/100 mL, 5 g/100 mL) were combined as reported in Table 1, taking into account real operating ranges. In addition, three replicates of the NOC_13_ batch (named NOC_13a_, NOC_13b_, and NOC_13c_) that combines the intermediate levels of the considered operating factors (i.e., temperature, 35 °C; pH 6.5; fat content, 2.55 g/100 mL), were performed to assess the reproducibility of FT-NIR measurements and MCR-ALS models.

Three failure batches (named FB_1_, FB_2_, and FB_3_) were also set up according to NOC_13_ operating conditions but forcing anomalies as follows: in FB_1_ only half the amount of rennet was added; in FB_2_ milk heating was turned off just after rennet addition; in FB_3_ only half the amount of CaCl_2_ was added.

### 2.2. Milk Preparation and Renneting

Pasteurized skimmed milk (Centrale del Latte di Milano, Milan, Italy; fat, 0.1 g/100 mL) was purposely combined with pasteurized milk cream (Centrale del Latte di Milano, Milan, Italy; fat, 35 g/100 mL) in order to reach the different fat concentrations imposed by the experimental plan (Table 1). Each mixture (150 mL) was kept under stirring on a magnetic plate for 12 h in a cold room (4 ± 1 °C). After reconditioning to 20 °C and CaCl_2_ addition (final concentration, 6 mM), pH was monitored, by using a previously calibrated pH-meter (mod. 3510; JENWAY, Dunmow, England), and adjusted to the desired value by the addition of a concentrated solution of citric acid (100 g/100 mL). Each sample was then heated and maintained at the desired operating temperature (±0.1 °C) by means of a thermostatic bath (Heidolph, MR Hei-Standard, Schwabach, Germany). After the addition of 52.5 µL liquid bovine rennet (75% chymosin, 25% rennin; Linea Rossa, 175 IMCU/mL, Caglificio Clerici, Cadorago, Como, Italy), the sample was stirred for 60 s before starting monitoring by FT-NIR spectroscopy and rheology.

### 2.3. FT-NIR Spectroscopy

Each milk sample (100 mL), prepared as previously reported, was maintained in a thermostatic bath (Heidolph, MR Hei-Standard, Schwabach, Germany) at the required temperature (30 °C, 35 °C, or 40 °C) and monitored for 30 min through a FT-NIR spectrometer (MPA, Bruker Optics, Milan, Italy) equipped with a transflectance optic probe (1 mm pathlength) inserted directly in the sample. Spectra were collected every minute over the 12,500–4000 cm^−1^ range, with a resolution of 8 cm^−1^, and 64 scans for both sample and background in order to obtain a good signal-to-noise ratio. The instrument control was managed by OPUS software (v. 6.0 Bruker Optics, Milan, Italy).

### 2.4. Rheological Behaviour

Milk renneting batches were monitored in continuous also by rheology measurements. In particular, a time curing test in oscillation was performed by means of a Physica MCR 300 rheometer (Anton Paar GmbH, Graz, Austria), controlled by the software Rheoplus/32 (v. 3.00, Physica Messtechnik GmbH, Ostfildern, Germany). Each milk sample (19 mL), prepared as previously reported, was poured in the concentric cylinders (CC27) of the rheometer heated at the desired temperature (30 °C, 35 °C, or 40 °C). Elastic (G′) and viscous (G″) modulus were measured each minute over a 30 min period, applying constant strain (0.01%) and frequency (1 Hz) values. Strain and frequency settings were chosen based on preliminary strain and frequency sweep tests carried out on both liquid and coagulated milk samples.

G′ values of each batch were modelled as a function of coagulation time, using the following sigmoid curve (Equation (1)) implemented in Table Curve software (v. 4.0, Jandel Scientific, San Rafael, CA, USA):

(1) $$y=a/\left(1+\exp\left(\frac{-\left(x-b\right)}{c}\right)\right)$$

In order to identify kinetic critical points during renneting—i.e., times related to the maximum rate, acceleration, and deceleration of the process—the first and second derivatives of the sigmoid functions were calculated. Afterwards, the times corresponding to maximum and minimum values of derivatives were extrapolated [18].

### 2.5. Data Analysis

FT-NIR spectra collected during milk coagulation trials were reduced in spectral range (12,500–5824 cm^−1^) and batch-wise pre-processed with Standard Normal Variate (SNV). Spectral region from 5823 to 4000 cm^−1^ was excluded due to high noise and signal saturation.

MCR-ALS analysis was performed by using a toolbox [22] implemented in MatLab v. 7.4 (MathWorks, Natick, MA, USA). Spectral data for NOC batches 1–12 were arranged in twelve **D** (M × N) sub-matrices, where the *M* rows correspond to the 30 spectra obtained at different renneting times and the *N* columns refer to the 1730 considered wavenumbers. Spectral data obtained from the three replicates of NOC_13_ and the failure batches (FB_1_, FB_2_, and FB_3_) were arranged in a **D** matrix composed of 6 independent sub-matrices, each referring to one of the 6 trials.

MCR-ALS allowed the decomposition of each **D** matrix into two sub-matrices, **C** (*M* × *F*) and **S**^T^ (*F* × *N*), named concentration and spectral profiles respectively (Equation (2)). C describes the *F* components affecting the modification of the *M* spectra over time, whereas **S**^T^ contains the *F* component variations with respect to the considered *N* wavenumbers. **E** (*M × N*) is the residual matrix.

**D** = **CS**^T^ + **E** (2)

Before applying MCR, the number of components (*F*) was defined by Principal Component Analysis (PCA). Then, the ALS optimization was performed by using a previously stated initial estimation of the spectral profiles [23].

The general steps of MCR-ALS are the following [24]:

1. Definition of the component number (*F*) for **D**.
2. Development of non-random initial estimates of either **C** or **S**^T^.
3. Given **D** and **S**^T^, least-squares calculation of C under given constraints.
4. Given **D** and **C**, least-squares calculation of **S**^T^ under given constraints.
5. Reconstruction of D as the product **CS**^T^.

The last three steps have been repeated until the quality in data reconstruction was satisfactory and convergence in the iterative optimization was achieved. The proper final concentration and spectral profiles were determined by using a stopping criterion based on the relative difference of the Lack of Fit percentage (LOF; Equation (3)), i.e., when the LOF difference in two consecutive iterative cycles was lower than 0.1% [25].

(3) $$LOF\;\left(\%\right)=100\times \sqrt{\frac{\sum_{ij}e_{ij}^{2}}{\sum_{ij}d_{ij}^{2}}}$$

In this equation, *e_ij_* is each *ij*th element of the residual matrix **E**, i.e., the related residual obtained from the difference between the input element and the MCR-ALS reproduction, and *d_ij_* is each *ij*th element of the **D** matrix. Non-negative concentration and unimodality constraints were imposed on concentration profiles to solve MCR-ALS ambiguities due to rotational and scale intensity.

The concentration profiles calculated by MCR-ALS were compared with the rheological results to assess the reliability of the approach. In particular, a Pearson correlation matrix was calculated for kinetic critical times extrapolated from the G′ curves and the time corresponding to the maximum value of the second MCR-ALS profile.

PCA-based MSPC charts were built using MCR-ALS concentration profiles obtained for the three replicates of NOC_13_ batch. The concentration profiles of NOC_13c_ and failure batches (FB_1_, FB_2_, and FB_3_) were then used to detect if each considered batch was in or out of control based on the MSPC charts previously built. The PCA-based MSPC charts [26] were developed calculating Hotelling’s T^2^ and Q statistics for the PCA models constructed with the MCR-ALS concentration profiles. The Hotelling’s T^2^ chart represents the estimated Mahalanobis distance from the centre of the PCA model. The Q-statistic chart analyses the residuals, i.e., the process variations not represented in the PCA model. Sensitivity (i.e., true positive rate) and specificity (i.e., true negative rate) were calculated to evaluate the reliability of each control chart.

All the chemometric analyses were performed by using MatLab v. 7.4 (MathWorks, Natick, MA, USA).

## 3. Results and Discussion

### 3.1. FT-NIR Spectra Interpretation

**Figure 1:**
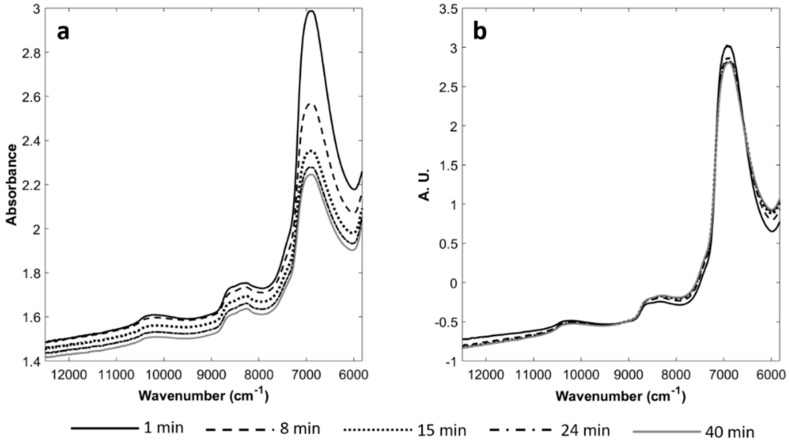
FT-NIR spectra collected for one milk renneting batch developed under normal operating conditions (NOC_13a_): temperature, 35 °C; milk pH, 6.5; fat content, 2.55 g/100 mL. (**a**) raw spectra; (**b**) Standard Normal Variate (SNV) pre-treated spectra.

The FT-NIR spectra collected during the renneting trials showed a clear increment in absorbance over time, highly affected by scattering effects due to physical changes of milk caused by coagulation [27,28], as well as to instrument characteristics. As an example, spectra trend during coagulation of batch NOC_13a_ is shown in Figure 1a. The main absorption band, observable at 6900 cm^−1^, is due to the combination of symmetric and asymmetric stretching of O-H bond of water, as already observed in previous works [14,17]. Other relevant bands at 10,800 and 8600 cm^−1^ are ascribable to the lipid C-H bonds [29].

After SNV pre-treatment (Figure 1b), it was possible to notice that the spectrum collected at the beginning of the process (just after 1 min) had a higher absorbance within the 12,000–9000 cm^−1^ range if compared to the spectra acquired from 8 min on. The opposite occurs between 9000 cm^−1^ and 7000 cm^−1^. In correspondence of O-H water symmetric and asymmetric stretching (6900 cm^−1^) the absorbance of the liquid milk resulted again higher than that of the spectra collected on coagulating milk. Similar spectra and trends were observed for all the performed experimental trials.

### 3.2. Principal Component Analysis (PCA)

**Figure 2:**
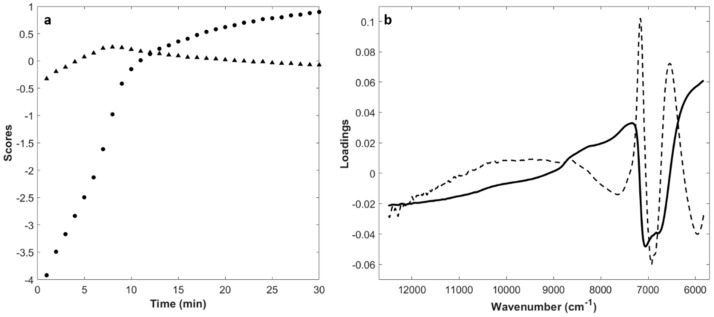
Principal component analysis results of the SNV transformed and centered FT-NIR spectra collected for one milk renneting batch developed under normal operating conditions (NOC_13a_): temperature, 35 °C; milk pH, 6.5; fat content, 2.55 g/100 mL. (**a**) plot of PC1 (●) and PC2 (▲) scores vs time; (**b**) PC1 (solid line) and PC2 (dashed line) loading plot.

Each dataset collected from a single milk renneting batch was explored by PCA after spectral range reduction and pre-treatment with SNV and mean-centering. The number of significant components *F* to be used in the following MCR-ALS analysis was chosen by inspecting scores, loadings, and explained variance of PCA models. Figure 2 shows, as an example, the trend of the first and second principal component (PC1 and PC2) scores vs. coagulation time and the corresponding loadings plot obtained for batch NOC_13a_. Similar results were obtained for all the NOC batches evaluated. PC1 explained at least 98.54% of variance and described changes occurring in milk during renneting. Actually, the PC1 scores vs. time presented a sigmoid-like distribution with a fast increase in score values up to 10 min of coagulation (●, Figure 2a). Then, the score increment rate decreased, reaching a steady state at the end of coagulation monitoring. At the beginning of the monitoring, samples were characterized by slightly negative values of PC2 (▲, Figure 2a); afterwards, they rapidly moved to a maximum in correspondence of the maximum increasing rate of PC1 (i.e., the maximum slope of PC1 curve), and then they decreased and stabilized. A very similar PC1 score trend was also observed for milk fermentation by Grassi et al. [17]. Moreover, Lyndgaard *et al*. [14] found a similar time-related distribution of PC1 scores for milk renneting processes, highlighting three coagulation phases: k-casein proteolysis, paracasein aggregation, and gel network formation. The intensity of PC1 (solid line) and PC2 (dashed line) loadings (Figure 2b) showed that the wavenumbers mainly responsible for score distribution are associated with the major bands already observed and commented for raw and SNV pre-treated spectra (Figure 1).

### 3.3. MCR-ALS Results for NOC Batches

Before performing MCR analysis, the selection of the proper number of components was performed through singular value decomposition (SVD algorithm on which PCA is based) by the ALS approach. Since two components were used to describe the process changes observed in PCA of mean-centered data, it is consistent that three components were found relevant by ALS applied to the not-centered data, because the rank decreases by one when mean-centering is performed [30].

After determining the number of significant components, it was necessary to choose the initial estimates for starting the MCR-ALS analysis. Three spectra were selected for each NOC batch by means of Pure Variable detection, which selects the purest column variables of a dataset based on the SIMPLISMA method [31]. In all cases, the pure spectra selected by the algorithm corresponded to signal collected (1) at the beginning of the process, (2) at the time of significant reduction in absorbance at 6900 cm^−1^, related to the end of the paracasein aggregation, and (3) at the end of the monitoring. The three selected spectra are thus linked to the different renneting phases, as already reported for yogurt coagulation [18].

The product **CS^T^** of the obtained models explained at least 99.93% of the data variance; LOF was lower than 0.44%, and the standard deviation of the residuals was lower than 0.0067.

**Figure 3:**
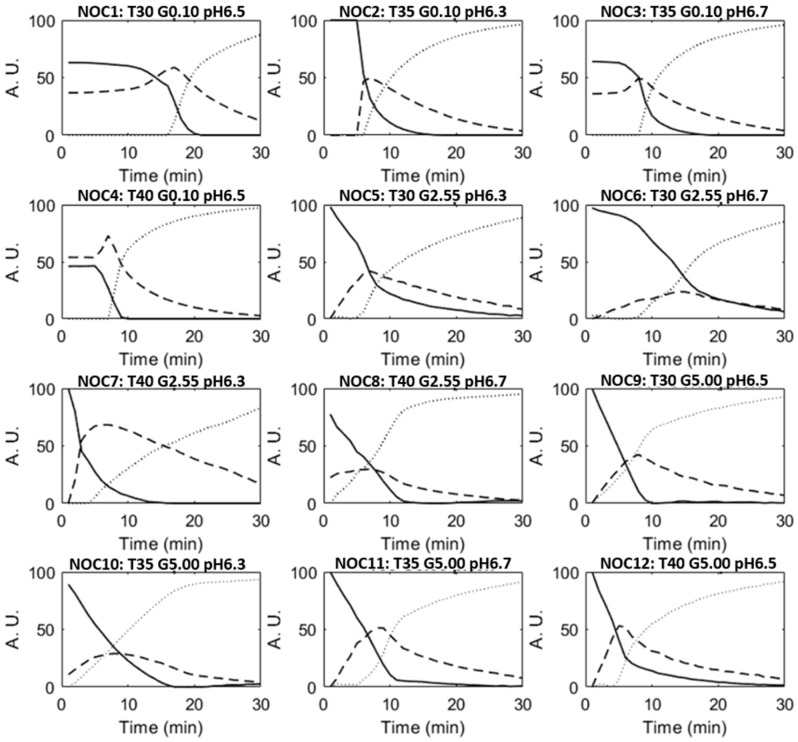
MCR-ALS concentration profiles of the milk renneting batches developed under normal operating conditions (NOC_1_-NOC_12_). Solid lines describe the liquid-like behavior of milk, dashed lines represent the transition phase of renneting, and dotted lines reflect the solid-like behavior of coagulated milk. See Table 1 for sample identification.

Figure 3 reports the MCR-ALS concentration profiles obtained for the 1–12 NOC batches of milk renneting. The trend of the three concentration profiles contains information about the well-known changes occurring during milk coagulation, already highlighted by the trend of PC1 and PC2 score values vs. renneting time and also confirmed by the rheological measurement results. In particular, the first MCR-ALS concentration profile (solid lines) had a sigmoidal shape in all the performed renneting trials, with higher values at the beginning of the process as already observed by Grassi et al. [18] for milk fermentation. Actually, the first concentration profile describes the primary phase of coagulation, which involves the k-casein proteolysis [14,32]. In this phase, no changes in the liquid-like structure of milk can be observed. Later, when the first aggregation of paracasein occurs, a steep decrease in the first MCR-ALS profile was observed, because milk was no more liquid. In the second phase of renneting, described by the second MCR-ALS concentration profile (dashed lines in Figure 3), the massive aggregation of the rennet-altered casein micelles occurs due to the glycomacropeptide detachment and the consequent loss of colloidal stability, thus forming chains and clusters [14,32]. Time corresponding to the maximum value of the second MCR-ALS profile can therefore be considered as the milk sol-gel transition point. Afterwards, coagulation enters the third phase, when the protein clusters grow until they form a continuous, three-dimensional gel network incorporating water and fat [14,33]. This phase is represented by the third MCR-ALS concentration profile (dotted lines in Figure 3), which had a sigmoidal shape opposite to the first profile. The three coagulation phases are highly interconnected and partially overlap over the coagulation time.

**Figure 4:**
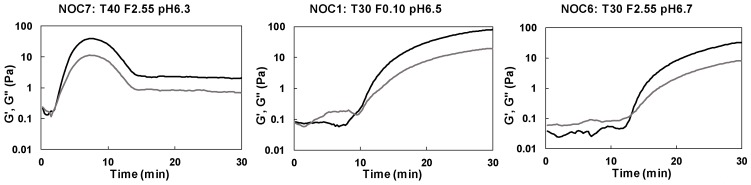
Time curing profiles of some of the evaluated milk renneting batches. Black and grey lines represent the elastic (G′) and viscous (G″) modulus, respectively. See Table 1 for sample identification.

The different shapes and timing of the concentration profiles were strictly related to the combination of the investigated processing factors, i.e., temperature, milk pH and fat content. As expected, milk coagulation resulted faster in trials performed at 40 °C with a pH value of 6.3, as evidenced by the advance transition time highlighted by the second concentration profile (dashed line) of NOC_7_ batch (sample ID: T40 F2.55 pH6.3). NOC_1_ (sample ID: T30 F0.10 pH6.5) and NOC_6_ (sample ID: T30 F2.55 pH6.7) batches showed a slower coagulation, due to the combination of low temperature (30 °C) and high pH values (6.5 and 6.7, respectively). These observations were also confirmed by the time curing profiles reported as an example in Figure 4, where the three renneting phases can be clearly distinguished. At the beginning, no changes in the liquid-like behaviour of milk were observed, as the enzymatic modification of casein micelles did not affect the elastic (G′) and viscous (G″) moduli trend. Then, an increase in both G′ and G″ values was registered, corresponding to the formation of a three-dimensional protein network incorporating water and fat. Even though the time curing profiles of all the performed trials showed a similar behaviour, each operative condition led to characteristic trends and shapes of G′ and G″ curves. In particular, for NOC_7_ batch (sample ID: T40 F2.55 pH6.3) the process was so fast that also the coagulum break was evident, with a decrease in G′ and G″ values after less than 10 min of renneting. Such a decrease corresponds to a strong whey syneresis and it was evident only for this sample. A similar behaviour of G′ values during milk renneting at pH6.1 was also reported by Ong et al. [34].

To better highlight the relationship between FT-NIR spectroscopy and rheology data, a Pearson correlation matrix was calculated for kinetic critical times extrapolated from G′ curves and the time corresponding to the maximum value of the second MCR-ALS profile. The latter resulted to be highly correlated (*r* = 0.96; *p* < 0.001) with the acceleration time of renneting calculated from the G′ curves (Table 2), indicating that both the parameters describe the sol-gel transition of milk. However, the transition times measured by FT-NIR spectroscopy occurred always few minutes before those calculated from rheological curves, thus demonstrating a higher sensitivity of the spectroscopic technique to the coagulation phenomena. This higher sensitivity of FT-NIR spectroscopy with respect to rheological data was reported also by Grassi et al. [17] for milk fermentation and it can be relevant for a better renneting control at industrial level.

These findings reveal that FT-NIR spectroscopy, combined with MCR-ALS, represents a robust approach for the description of milk renneting under different cheese-making conditions. Indeed, this approach was able to distinguish the three-main coagulation phases, no matter the operating temperature, and the milk pH and fat content.

### 3.4. MCR-ALS Results for NOC_13_ and FB Batches

**Figure 5:**
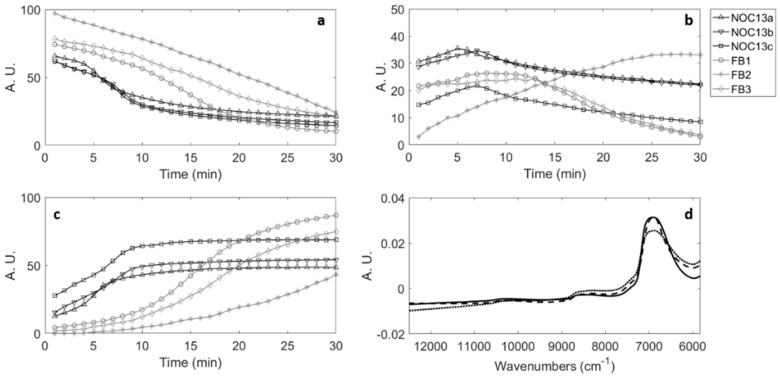
MCR-ALS results for the three replicates of NOC_13_ (normal operating conditions) batch and for the three failure batches (FB): (**a**) concentration profiles of the liquid-like behavior of milk; (**b**) concentration profiles of the transition phase of renneting; (**c**) concentration profiles of the solid-like behavior of coagulated milk; (**d**) spectral profiles of liquid-like behavior of milk (solid line), transition phase (dashed line) and solid-like behavior of milk (dotted line).

The reliability of the proposed FT-NIR method based on MCR-ALS models to assess possible milk renneting failures was verified using the spectral data matrix containing FT-NIR data collected from the three replicates of the NOC_13_ batch and the three failure trials FB_1_, FB_2_ and FB_3_. Figure 5 shows the three MCR-ALS concentration profiles obtained for the six considered batches and their corresponding spectral profiles. The **CS^T^** product explained 99.99% of the data variance; LOF was 0.63%, and the standard deviation of the residuals was lower than 0.0063. The concentration profiles obtained for the three replicates of NOC_13_ batch (reported in black in Figure 5a–c) were almost overlapped and the small differences observed should be considered as the expected variability in a milk renneting process carried out with different lots of raw materials.

The concentration profiles of NOC_13_ replicates showed the same trends already observed for the NOC batches 1–12, thus, also in this case, the sol-gel transition time can be extrapolated as the time corresponding to the maximum value of the second profile. MCR-ALS profiles for FB batches 1–3 (reported in grey in Figure 5a–c) were clearly separated in timing and shape from the profiles of NOC_13_ replicates, according to the imposed failures in the process. The pure spectral profiles obtained (Figure 5d) are representative of the different coagulation phases, as already observed for NOC_1:12_ (data not shown): the solid-line profile represents the liquid-like behaviour of milk; the dotted-line profile represents the solid-like behaviour of coagulated milk; the dashed-line profile stands for the transition phase.

### 3.5. MSPC Charts on MCR-ALS Results for NOC_13_ and FB Batches

**Figure 6:**
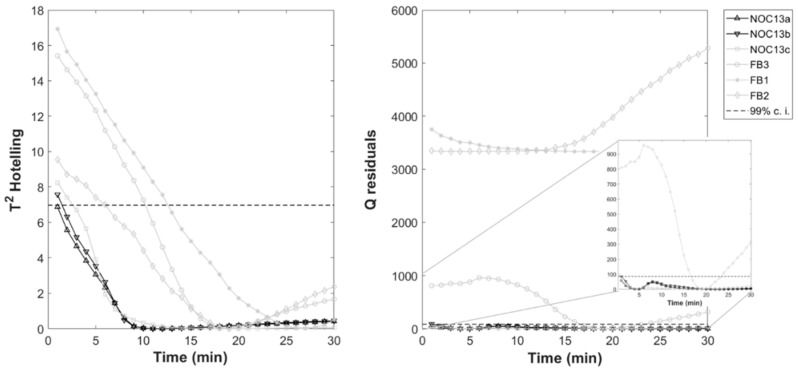
Multivariate Statistical Process Control (MSPC) charts for the three replicates of the milk renneting batch NOC_13_ (in-control) and for the three failure batches FB (off-control): (**a**) Hotelling’s T^2^; (**b**) Q-statistic. Dashed line represents the 99% confidence interval (c.i.).

PCA-MSPC charts were built applying PCA to the three MCR-ALS concentration profiles calculated for the replicates “a” and “b” of NOC_13_ batch. The first principal component was considered, accounting for 80% of the variance. A 99% confidence interval was considered in order to calculate the chart limits (dashed lines in Figure 6). Then, the MCR-ALS concentration profiles of replicate “c” of NOC_13_ batch were also projected into the PCA model, as well as the profiles calculated for the failure batches (FB_1_, FB_2_, and FB_3_). The relative Hotelling’s T^2^ and Q statistics were calculated and implemented in the control charts. The NOC_13c_ batch resulted in-control over all the renneting time, i.e., within the calculated confidence interval. On the contrary, the FB batches showed off-control values of T^2^ up to 12 min after the beginning of the trials and off-control values of Q residuals for most of the sampling points.

The goodness of the built charts was evaluated also by sensitivity and specificity values. T^2^ chart showed quite poor specificity (19%) due to the in-control values reached by the tested batches with coagulation progress; on the contrary, sensitivity was high (95%). These results can be due to the fact that the variables used, i.e., the concentration profiles, are highly correlated, thus monitoring process compliance through T^2^ based on the first PC could be not sufficient [26]. It is therefore advisable to refer also to the Q-statistic [35]. In this case study, Q residual chart gave both high specificity (94%) and sensitivity (100%), suggesting the reliability of this approach for the monitoring of milk renneting. By the combination of T^2^ and Q control charts, it was possible to detect the in-control tested batch (NOC_13c_) and to distinguish failure batches just from the first minutes of the process. Similarly, de Oliveira et al. [30] concluded that the combination of T^2^ and Q charts gave specificity and sensitivity results more reliable than their single check when applying MSPC to synthetic commercial gasoline distillation.

## 4. Conclusions

The work demonstrated that coupling FT-NIR spectroscopy with MCR-ALS data elaboration allows the development of a useful tool for the in-line control of milk renneting during cheese manufacturing. The very innovative approach suggested for the implementation of MSPC charts is able to detect possible coagulation failures from the first minutes of the process. This is of fundamental importance for modern dairy industries, because of the urgent needs for automation in order to improve product quality and production yields. This kind of industrial control systems perfectly fit with the Industry 4.0 roadmap towards a fully digital enterprise.

## Figures and Tables

**Table 1:** Milk renneting batches set up under normal operating conditions (NOC) for the development of a process monitoring tool. * NOC_13_ conditions were performed in three replicates (NOC_13a_, NOC_13b_, and NOC_13c_).

Batch	Sample ID	Temperature (°C)	Fat Content (g/100 mL)	pH
NOC_1_	T30 F0.10 pH6.5	30	0.10	6.5
NOC_2_	T35 F0.10 pH6.3	35	0.10	6.3
NOC_3_	T35 F0.10 pH6.7	35	0.10	6.7
NOC_4_	T40 F0.10 pH6.5	40	0.10	6.5
NOC_5_	T30 F2.55 pH6.3	30	2.55	6.3
NOC_6_	T30 F2.55 pH6.7	30	2.55	6.7
NOC_7_	T40 F2.55 pH6.3	40	2.55	6.3
NOC_8_	T40 F2.55 pH6.7	40	2.55	6.7
NOC_9_	T30 F5.00 pH6.5	30	5.00	6.5
NOC_10_	T35 F5.00 pH6.3	35	5.00	6.3
NOC_11_	T35 F5.00 pH6.7	35	5.00	6.7
NOC_12_	T40 F5.00 pH6.5	40	5.00	6.5
NOC_13_ *	T35 F2.55 pH6.5	35	2.55	6.5

**Table 2:** Milk renneting: comparison of time corresponding to the maximum value of the second MCR-ALS concentration profile calculated from FT-NIR data (CP2) and time corresponding to acceleration in elastic modulus (G′) increase extrapolated from time curing curves (AT_G′).

Batch	Sample ID	CP2 (min)	AT_G′ (min)
NOC_1_	T30 F0.10 pH6.5	14.5	18.9
NOC_2_	T35 F0.10 pH6.3	6.0	7.9
NOC_3_	T35 F0.10 pH6.7	9.0	13.3
NOC_4_	T40 F0.10 pH6.5	8.0	11.2
NOC_5_	T30 F2.55 pH6.3	7.3	10.0
NOC_6_	T30 F2.55 pH6.7	17.0	19.4
NOC_7_	T40 F2.55 pH6.3	6.5	8.8
NOC_8_	T40 F2.55 pH6.7	7.0	9.5
NOC_9_	T30 F5.00 pH6.5	8.3	13.1
NOC_10_	T35 F5.00 pH6.3	8.0	11.0
NOC_11_	T35 F5.00 pH6.7	8.5	13.7
NOC_12_	T40 F5.00 pH6.5	5.5	6.8
NOC_13a_	T35 F2.55 pH6.5	5.8	8.9
NOC_13b_	T35 F2.55 pH6.5	7.5	9.8
NOC_13c_	T35 F2.55 pH6.5	7.0	9.4
